# Supplementary material for: A predictive model of joint dynamics and ground reaction force using only leg length, body mass, and walking cadence
Source: PLoS One. 2026 Jan 2;21(1):e0338041. doi: 10.1371/journal.pone.0338041 (PMC12758755; doi:10.1371/journal.pone.0338041)
Supplement: Attachment — Submitted filename: response letter.docx [file pone.0338041.s002.docx]

**Response Letter**

*We appreciate the editor’s and reviewers effort on this manuscript. The revisions have been highlighted in the revised manuscript (marked version) with "yellow background". For convenient review, the page numbers of the point-to-point response and revision list to the reviewers are cited below:*

*Response to editors in Pages 1-3.*

*Response to Reviewer 1 in Page 4.*

*Response to Reviewer 2 in Pages 4-10.*

***Response to editor***

1. Please ensure that your manuscript meets PLOS ONE's style requirements, including those for file naming. The PLOS ONE style templates can be found at

https://journals.plos.org/plosone/s/file?id=wjVg/PLOSOne_formatting_sample_main_body.pdf and

https://journals.plos.org/plosone/s/file?id=ba62/PLOSOne_formatting_sample_title_authors_affiliations.pdf.

***Response****: We appreciate the editors careful suggestions. We have carefully reviewed the PLOS ONE style requirements and have now formatted our manuscript and renamed our files to be fully compliant.*

*The revised files have been uploaded to the submission system.*

2. Please note that PLOS One has specific guidelines on code sharing for submissions in which author-generated code underpins the findings in the manuscript. In these cases, we expect all author-generated code to be made available without restrictions upon publication of the work. Please review our guidelines at https://journals.plos.org/plosone/s/materials-and-software-sharing#loc-sharing-code and ensure that your code is shared in a way that follows best practice and facilitates reproducibility and reuse.

***Response****: We appreciate the editors careful suggestions. We fully understand and support the journal's commitment to transparency and reproducibility, which aligns with our own academic values.*

*We wish to bring to your attention that the core algorithm presented in this manuscript is the subject of a currently pending patent application. As you are undoubtedly aware, public disclosure of the source code at this stage would irrevocably compromise the novelty of the invention and jeopardize the patent application process.*

*Therefore, we are legally constrained from sharing the code publicly until the patent process is finalized. However, we are committed to providing maximum transparency within these legal boundaries and would like to propose the following plan to facilitate review and future reproducibility:*

*Upon successful granting of the patent (or at a stage where public disclosure is legally permitted), we unconditionally commit to depositing the entire codebase in a permanent repository with a DOI and an open-source license at the followed link:*

<https://github.com/zhaoxiaohuan13/predictive-model-of-joint-dynamics-and-ground-reaction-force>

3. Thank you for stating the following financial disclosure:

[This work was supported by:

China Postdoctoral Science Foundation [Grant No. GZB20240603]

Author: [H.Zhao]

Funder: China Postdoctoral Science Foundation

URL: http://jj.chinapostdoctor.org.cn

National Key Research and Development Program of China [Grant No. 2021YFE0203400]

Author: [J.Cao]

Funder: Ministry of Science and Technology of the People's Republic of China

URL: http://www.most.gov.cn

Mainland-Hong Kong Joint Funding Scheme (MHKJFS) [Project No. MHP/043/20]

Author: [W.H. Liao]

Funder: Innovation and Technology Commission, Hong Kong Special Administrative Region

URL: https://www.itc.gov.hk

The Chinese University of Hong Kong [Project ID: 3130174]

Author: [H.Zhao]

Funder: The Chinese University of Hong Kong

URL: https://www.cuhk.edu.hk

Direct Grant for Research 2024/2025 [Project ID: 4055254]

Author: [H.Zhao]

Funder: The Chinese University of Hong Kong

URL: https://www.cuhk.edu.hk].

Please state what role the funders took in the study. If the funders had no role, please state: ""The funders had no role in study design, data collection and analysis, decision to publish, or preparation of the manuscript.""

If this statement is not correct you must amend it as needed.

Please include this amended Role of Funder statement in your cover letter; we will change the online submission form on your behalf.

***Response****: "The funders had no role in study design, data collection and analysis, decision to publish, or preparation of the manuscript."*

4. When completing the data availability statement of the submission form, you indicated that you will make your data available on acceptance. We strongly recommend all authors decide on a data sharing plan before acceptance, as the process can be lengthy and hold up publication timelines. Please note that, though access restrictions are acceptable now, your entire data will need to be made freely accessible if your manuscript is accepted for publication. This policy applies to all data except where public deposition would breach compliance with the protocol approved by your research ethics board. If you are unable to adhere to our open data policy, please kindly revise your statement to explain your reasoning and we will seek the editor's input on an exemption. Please be assured that, once you have provided your new statement, the assessment of your exemption will not hold up the peer review process.

***Response****: Thank you for your reminder regarding PLOS One's data availability policy.*

*We confirm that we will adhere to the journal's open data policy. Our complete dataset will be made freely accessible upon publication of the manuscript.*

*We have now finalized our data sharing plan. We are in the process of preparing the data and documentation and will complete the deposition promptly. The corresponding explanation has been added in the revised manuscript at page 22 line 411 as follows:*

The data will be held in a public repository, and URLs (<https://github.com/zhaoxiaohuan13/predictive-model-of-joint-dynamics-and-ground-reaction-force>) will only be available after acceptance.

*We appreciate the guidance and will ensure this does not delay the publication timeline.*

5. If the reviewer comments include a recommendation to cite specific previously published works, please review and evaluate these publications to determine whether they are relevant and should be cited. There is no requirement to cite these works unless the editor has indicated otherwise.

***Response****: We confirm that we have noted this policy. After a thorough examination of all review comments, we found that the reviewers did not suggest any specific publications for citation. As such, this requirement is not applicable for this revision.*

***Reviewers' comments:***

Reviewer's Responses to Questions

Comments to the Author

1. Is the manuscript technically sound, and do the data support the conclusions?

The manuscript must describe a technically sound piece of scientific research with data that supports the conclusions. Experiments must have been conducted rigorously, with appropriate controls, replication, and sample sizes. The conclusions must be drawn appropriately based on the data presented.

Reviewer #1: Yes

Reviewer #2: Yes

2. Has the statistical analysis been performed appropriately and rigorously?

Reviewer #1: Yes

Reviewer #2: Yes

3. Have the authors made all data underlying the findings in their manuscript fully available?

The PLOS Data policy requires authors to make all data underlying the findings described in their manuscript fully available without restriction, with rare exception (please refer to the Data Availability Statement in the manuscript PDF file). The data should be provided as part of the manuscript or its supporting information, or deposited to a public repository. For example, in addition to summary statistics, the data points behind means, medians and variance measures should be available. If there are restrictions on publicly sharing data—e.g. participant privacy or use of data from a third party—those must be specified.

Reviewer #1: Yes

Reviewer #2: No

4. Is the manuscript presented in an intelligible fashion and written in standard English?

PLOS ONE does not copyedit accepted manuscripts, so the language in submitted articles must be clear, correct, and unambiguous. Any typographical or grammatical errors should be corrected at revision, so please note any specific errors here.

Reviewer #1: Yes

Reviewer #2: Yes

5. Review Comments to the Author

Please use the space provided to explain your answers to the questions above. You may also include additional comments for the author, including concerns about dual publication, research ethics, or publication ethics. (Please upload your review as an attachment if it exceeds 20,000 characters)

***Response to Reviewer 1***

Reviewer #1: I consider that the study is performed in accordance with requirements. The methodology is properly explained. The study claims it is an easy to measure technique.

I specifically appreciated the high applicability of the described method.

***Response****:We sincerely thank the reviewer for the encouraging feedback and recognition of our work’s novelty and effectiveness. We appreciate your acknowledgment of the clear presentation of our methodology and the value of our dynamic federated learning strategy in addressing data limitations, individual variations, and privacy concerns. We will continue to refine it for broader clinical application.*

***Response to Reviewer 2***

Reviewer #2: 1. Overall Evaluation

This manuscript presents a predictive model for estimating sagittal-plane gait dynamics (joint angles, joint moments, and ground reaction forces) based on only three input variables: leg length, body mass, and cadence. The minimal data requirements make this model potentially attractive for use in low-resource clinical or field settings. The mathematical structure is well developed and the model is validated against experimental data from healthy participants.

However, several conceptual and practical limitations significantly affect the model’s generalizability and translational value. These limitations are either insufficiently discussed or entirely unacknowledged in the manuscript. The authors are encouraged to clarify the intended scope of the model and to openly address its current boundaries, particularly regarding its clinical utility.

***Response****:We sincerely thank the reviewer for his/her positive feedback on the mathematical development of our model and for raising these critical points regarding its generalizability and translational value. We agree entirely that a more transparent and thorough discussion of the model's limitations is essential. We have significantly revised the manuscript to address these concerns.*

2. Strengths

- The model is simple, uses non-invasive and easily obtainable inputs, and has potential for low-cost deployment.

- It captures the full gait cycle, including both single and double support phases.

- The mathematical derivations are clearly presented and fully reproducible in principle.

- The inclusion of mass transfer during the double support phase is a novel and elegant feature for ensuring continuity of force and moment trajectories.

- Comparative performance is presented relative to existing models in the literature.

***Response****:We sincerely thank the reviewer for the encouraging feedback and recognition of our work’s novelty and effectiveness.*

3. Major Limitations

3.1. Assumption of gait symmetry and cycle periodicity

The model assumes perfect left–right symmetry and step-to-step periodicity. While the authors briefly acknowledge that human gait is inherently variable, they do not fully discuss the practical implications of this assumption. Gait asymmetry and temporal variability are not only normal but often clinically informative—especially in populations with stroke, Parkinson’s disease, or orthopedic impairments. The assumption of symmetry limits the model’s applicability to pathological gait or real-world functional assessments. This point should be discussed in terms of how it restricts the model’s diagnostic and rehabilitative utility.

***Response****:We thank the reviewer for this exceptionally insightful and critical comment. We completely agree that the assumption of perfect gait symmetry and periodicity is a significant limitation of our current model, and we appreciate the reviewer's clear articulation of how this restricts the model's utility in clinical and pathological contexts. We have revised the manuscript to address this point.*

*The corresponding supplement has been added in the revised manuscript in “Section 4. Discussions .”(page20 line 382-385) as follows:*

The model’s reliance on perfect symmetry and periodicity overlooks natural and clinically meaningful gait asymmetries, making it unsuitable for pathology assessment or rehabilitation monitoring, thus it is limited to approximating sagittal-plane biomechanics in healthy gait.

*We hope the reviewer will satisfy with the revision.*

3.2. Use of cadence as the primary dynamic input

The model relies on cadence as the only time-dependent input variable. Although cadence is easy to measure, it is not a specific or sensitive indicator of gait pathology. Cadence changes may reflect alterations in walking speed, fatigue, or motivation, but not necessarily impairment. The manuscript does not address this limitation, nor does it review the limited clinical value of cadence alone as a predictor of gait quality. This is a significant conceptual gap that should be acknowledged and discussed.

***Response****: We thank the reviewer for this crucial insight. We completely agree that cadence alone is a non-specific measure. Our reliance on it was primarily driven by the goal of model simplicity, but we acknowledge that this choice comes at the cost of clinical specificity.*

*We have added a new paragraph in the“Section 4. Discussions .”(page20-21 line 385-390) as follows:*

In addition, relying on cadence as the sole temporal input oversimplifies gait, since cadence is influenced by factors such as speed, fatigue, and environment, limiting the model’s diagnostic specificity. The model should therefore be viewed as a functional estimator of overall gait mechanics, with future work focusing on incorporating more specific biomarkers to improve clinical relevance.

In addition, the manuscript does not address how cadence should be measured outside of laboratory environments. To ensure ecological applicability, the authors should recommend validated mobile or wearable tools, define acceptable accuracy thresholds, and consider performing a validation comparing cadence from reference systems (e.g., VICON) to field-based methods (e.g., inertial sensors or apps). Without this, the practical deployment of the model remains limited.

***Response****: We thank the reviewer for raising this critical point regarding the ecological measurement of cadence. We agree entirely that for the model to be deployed reliably, the method of obtaining its input must be standardized and validated, even when using simple tools.*

*While cadence can indeed be measured with minimal equipment (e.g., a stopwatch), the corresponding supplement has been added in the revised manuscript in “Section 2.2 Predictive joint angle modeling”(page 6 line 134-135) as follows:*

it can be measured by the assessor using a stopwatch to record a 60-second interval, counting the number of steps (initial contacts of the same foot) within that period,

*We hope the reviewer will satisfy with the revision.*

3.3. Use of a theoretical or ideal gait reference

The model appears to be designed as a generator of an idealized gait profile, which could serve as a target or reference for a given individual based on anthropometry and cadence. However, in clinical rehabilitation, the goal is not to restore a normative gait pattern but to optimize functionality, safety, and energy efficiency, which may involve accepting asymmetry or compensation. The authors suggest this use case but do not critically examine its limitations. The potential mismatch between theoretical predictions and real-world therapeutic goals should be addressed.

***Response****: We sincerely thank the reviewer for this profoundly insightful comment. We completely agree with this perspective and appreciate the opportunity to clarify the intended role of our model in a clinical context.*

*The corresponding supplement has been added in the revised manuscript in “Section 4. Discussions .”(page21 line 391-395) as follows:*

The model generates an idealized, symmetric pattern, whereas therapy may aim to optimize safety, reduce pain, or improve energy efficiency, potentially by leveraging compensatory strategies that appear as asymmetries. Clinicians may use the model's output not as a goal but as a comparator. Future work will focus on integrating this model with functional outcome measures.

*We hope the reviewer will satisfy with the revision.*

3.4. Lack of accessible implementation contradicts the model’s purpose

Although the model is mathematically well-documented and could be implemented using open-source environments such as R or Python, the authors do not provide any source code or mention future code availability. This omission limits reproducibility and transparency. More importantly, it contradicts the model’s stated goal of providing a simple and accessible alternative to expensive lab-based motion analysis. If the method is to be truly usable in low-resource settings, the implementation must also be openly available.

The authors should share the source code in a public repository (e.g., GitHub, Zenodo, OSF) using an open-access license and a programming language compatible with widespread use. This would allow researchers and clinicians to reproduce the results, adapt the model to specific populations, and ensure consistency in future validations.

***Response****: We thank the reviewer for his/her strong endorsement of our model's mathematical foundation and for underscoring the importance of code sharing for reproducibility and accessibility. We deeply agree with these principles in spirit.*

*However, we are in a challenging position regarding the public release of the source code. The implementation of this model is directly tied to a pending patent application, where the specific codebase is considered a trade secret and part of the intellectual property under protection. Public disclosure at this stage would jeopardize the patent process.*

*While we cannot share the exact source code, we are unequivocally committed to enabling full reproducibility and facilitating the model's use. To this end, we have taken the following substantive and immediate actions to address the reviewer's concerns as thoroughly as possible:*

*In direct response to this comment and in alignment with journal policy, we will publicly deposited the complete experimental dataset used to train and validate our model. This includes all raw kinematic, kinetic, and anthropometric data from our healthy participant cohort. The data will available once the manuscript is accepted. The corresponding explanation has been added in the revised manuscript at page 22 line 420 as follows:*

The data will be held in a public repository, and URLs (<https://github.com/zhaoxiaohuan13/predictive-model-of-joint-dynamics-and-ground-reaction-force>) will only be available after acceptance.

*Once the patent is issued, the code will also be added in to this repository.*

*We believe that the immediate public release of our data, combined with a complete algorithmic blueprint and a commitment to future code release, provides a robust path for verifying our findings and allows the community to build upon our work without delay. We are grateful for the reviewer's understanding.*

4. Formatting and Presentation

- Figures: Figures are clearly referenced and support the validation process effectively. However, captions should be expanded to clarify curve identities, sample size, and units.

***Response:*** *We thank the reviewer for his/her positive feedback and for this constructive suggestion. We had expanded some figure captions.*

*The supplement of Fig.1 has been added in the revised manuscript in “Section2.2 Predictive gait kinetics modeling.”(page 7 line 150-158) as follows:*

The gray circle represent the trunk with the pelvis. The gait cycle is initiated at left heel contact (0%) and is normalized to 100%. The diagram depicts the double support phases and single support phases. The first double support phase (0-12%): the period when both the feet is in contact with the ground (for example, the right leg is the trailing leg); The first single support phase (13-50%): the period when only one foot is on the ground (the right leg is swing, and left foot contact the ground); The second double support phase (51-62%): the period when both the feet is in contact with the ground (the right leg is the dominant leg). The first single support phase (63-100%): The period when only one foot is on the ground (the right leg is supporting, and right foot contact the ground).

*The supplement of Fig.6 has been added in the revised manuscript in “Section2.2 Predictive gait kinetics modeling.”(page 18 line 343-344) as follows:*

The measurement moment in the figure is obtained from the walking trials of the corresponding subject in the experiment.

*We hope the reviewer will satisfy with the revision.*

- Tables: Table 5 is difficult to interpret due to formatting complexity. It should be restructured for clarity, possibly in landscape format or as a supplementary file.

***Response:*** *We thank the reviewer for this helpful feedback. We agree that the previous formatting of Table 5 was complex and hindered readability. Following the reviewer's suggestion, we have converted Table 5 to landscape orientation within the main manuscript. It can be seen in the revised manuscript in “4. Discussions”(page 19-20 line 375) as follows:*

| Ref | Required sensors | Models/  Methods | Joint angle | | Joint moment | | anterior-posterior GRF | | Vertical GRF | |
| --- | --- | --- | --- | --- | --- | --- | --- | --- | --- | --- |
|  |  |  | rRMSE/% | ρ | rRMSE/% | ρ | rRMSE/% | ρ | rRMSE/% | ρ |
| Dorschky et al. 2019  [31] | 7 IMUs at pelvis, thighs, shanks, and feet | 7-segment musculoskeletal model | 8.8-21.9 | 0.96-1 | 14.4-27.1 | 0.76-0.95 | 9.7 | 0.95 | 9.6 | 0.95 |
| Lee et al. 2020 [32] | 1 IMU at sacrum | ANN | / | / | 11.4-24.08 | 0.20-0.77 | 9.21 | 0.88 | 6.7 | 0.93 |
| Li et al. 2021  [33] | 2 IMUs at shanks | 7-segment skeletal model | 11.5-26.1 | / | 20.4-29 | / | 3.9% | / | 12.2% | / |
| Ripic et al. 2022[34] | A single azure kinetic sensor | musculoskeletal model | 6.7-16.4 | 0.488-0.876 | / | / | 4.1 | 0.889 | 2.9 | 0.94 |
| Hossain et al. 2023[35] | 4 IMUs at thigh, shank, and foot  3 electronic goniometers at hip, knee and ankle | Kinetics-FM-DLR-Ensemble-Net | / | / | 5.59-6.06 | 0.765-0.868 | 3.77 | 0.922 | 5.49 | 0.957 |
| Simonetti et al. 2024[36] | 5 IMUs at pelvis, thigh, shank and feet | EMG-driven musculoskeletal model | / | 0.51-0.66 | 32 | 0.67 | / | 0.46 | / | 0.86 |
| Liew et al. 2025[37] | Walking speed measured by meter and second chronograph | Statistical model | 9.85-18.96 | 0.88-0.97 | 11.07-17.26 | 0.83-0.96 | 6.63 | 0.96 | 6.15 | 0.99 |
| This study | Walking cadence measured by second chronograph | 2 and3 -segment skeletal model | 7.38-9.84 | 0.97-0.99 | 13.06-20.95 | 0.84-0.93 | 11.83-12.93 | 0.92-0.93 | 9.99-11.05 | 0.93-0.94 |

- Symbols and notation: A glossary or table of variables would improve clarity and help readers follow the mathematical models more easily.

***Response****: We thank the reviewer for his/her thoughtful suggestion regarding the clarification of notation. Upon review, we note that the mathematical models in our manuscript primarily use standard biomechanical variables (e.g., L for length, m for mass, θ for joint angles) which are clearly defined upon first use in the text. Furthermore, there is only one abbreviated term (GRF for Ground Reaction Force) used throughout the manuscript, which is also defined at its first occurrence.*

*Therefore, to avoid unnecessary redundancy and maintain a concise format, we have not created a separate nomenclature table. Instead, we have ensured that all variables and the single acronym are explicitly defined inline within the manuscript to guarantee clarity for the reader.*

*We hope this approach is acceptable. Thank you again for raising this point.*
