# Supplementary material for: A predictive model of joint dynamics and ground reaction force using only leg length, body mass, and walking cadence
Source: PLoS One. 2026 Jan 2;21(1):e0338041. doi: 10.1371/journal.pone.0338041 (PMC12758755; doi:10.1371/journal.pone.0338041)
Supplement: Attachment — Submitted filename: response letter - R2.docx [file pone.0338041.s003.docx]

**Response Letter**

*We appreciate the editor’s and reviewers effort on this manuscript. The revisions have been highlighted in the revised manuscript (marked version) with "yellow background". For convenient review, the page numbers of the point-to-point response and revision list to the reviewers are cited below:*

*Response to editors in Pages 1.*

*Response to Reviewer 1 in Page 1.*

*Response to Reviewer 2 in Page 1.*

*Response to Reviewer 3 in Pages 1-8.*

***Response to editor***

The paper has been revised by three experts in the field. The manuscript has been recognied good by all the reviewers however some clarifications are needed. Please respond to the reviewers concerns point-by-point.

***Response****: We appreciate the editors careful suggestions. The point-by-point respond to reviewers have been listed in this letter.*

***Reviewers' comments:***

***Response to Reviewer 1***

Reviewer #1: The manuscript is in good shape for publishing. The questions of the reviewers were properly answered.

***Response****: We thank the reviewer for his/her helpful suggestions to improve this manuscript.*

***Response to Reviewer 2***

Reviewer #2: I would like to thank the authors for the detailed responses to my comments. I think the manuscript is now suitable for publication. Congrats!

***Response****: We sincerely thank the reviewer for the encouraging feedback and recognition of our work.*

***Response to Reviewer 3***

Reviewer #3: The study of Zhao and colleagues aims to develop a minimum input model for joint angle and GRF estimation based on a bipedal mechanism that switches between two subsystems, i.e., one related to the double support phase and one to the single support phase. Overall, the research topic is valid; however, many concerns are present in the paper, and I list below my main points.

***Response****: We would like to sincerely thank Reviewer #3 for their thorough review and valuable feedback on our manuscript. We appreciate the reviewer's acknowledgment of the validity of our research topic. The comments and concerns raised are highly insightful and have provided us with an excellent opportunity to significantly improve the quality of our work.* *In response to the reviewer's points, we have undertaken a comprehensive revision of the manuscript. The major modifications are listed point-by-point as follows:*

MAIN ISSUES
1. In the methods section, for the Fourier approximation, the authors must specify how many harmonics they considered in the model and how they tuned the coefficients. Although this can later be inferred from Table 10, it should be clearly reported in the methods section.

***Response****: We thank the reviewer for this excellent suggestion. We agree that the methodology should be self-contained and clearly stated. In response, we have revised the Methods section (specifically, in the subsection titled "[2.2 Predictive joint angle modeling]") to explicitly include the details of the Fourier approximation. The corresponding supplement has been added to page 7 line 138-145 as follows:*

Since *n* is the number of harmonics, it was determined based on a preliminary analysis which aimed to capture the essential dynamics of the signal while avoiding overfitting as illustrated in our previous research [28]. For thigh and knee joints, the first three harmonics were selected, while for ankle joint, the first four harmonics were selected, which provided an optimal balance between reconstruction accuracy and model complexity. In addition, the coefficients of the Fourier series “*B_j_*” were tuned by linear least-squares regression to minimize the error between the approximated signal and the original input data as demonstrated in [28].

*We hope the reviewer will satisfy with the revision.*

2. A major problem concerns the correspondence between the equations and Figures 2 and 3. The terms used in the equations are not clearly indicated in the figures, which makes the manuscript confusing and difficult to interpret. For instance, in Equation 3, the authors refer to the double support figure (Fig. 3), but they use the terminology Iand i-th, which seems more appropriate for Figure 2. Moreover, Equation 3 shows only two segments. This is just one example, but in general, there is a lack of consistency between the quantities shown in the figures and those indicated in the equations (consider also the masses of the segments). As a result, it is difficult to verify and check the equations against the figures.

***Response****: We sincerely thank the reviewer for this crucial observation. We fully acknowledge that the inconsistency between the equations and the figures was a significant weakness that hindered the clarity and verifiability of our methodology. We have thoroughly revised and explained both Equation 3 and Equation 4, and correspondingly adjust the two equations near the Fig 2 . The revisions are shown in the revised manuscript as follows:*

*at page 7 line 153-155:*

*M*_h_ represents the moment at hip for the supporting leg, *M*_h2_ represents the moment at hip for the swing leg, *M*_a_ represents the moment at ankle, and *M*_k_ represents the moment at knee.

*at page 9-10 line 178-188:*

In the Cartesian coordinate system with the foot in the stance phase as the origin, the Cartesian absolute angles are used to represent the centroid position and velocity of the standing leg.

|  | $\left[\begin{matrix} x{}_{i} \\ y{}_{i} \end{matrix}\right]=\left[\begin{matrix} \sum_{i=0}^{i-1} l{}_{i-1}\text{sin}\theta{}_{i-1} \\ \sum_{i=0}^{i-1} l{}_{i-1}\text{cos}\theta{}_{i-1} \end{matrix}\right]+\left[\begin{matrix} p{}_{i}\text{sin}\theta{}_{i} \\ p{}_{i}\text{cos}\theta{}_{i} \end{matrix}\right]$ | （3） |
| --- | --- | --- |

where $x_{i}$ and$y_{i}$ represent the horizontal and vertical displacement of the centroid of the *i* th segment, $l_{i-1}$ represents the length of the (*i*-1) th segment, $p_{i}$ represents the distance between the centroid and the starting point of the *i* th segment, and $\theta_{i}$ represents the angle between the *i* th segment and the vertical direction, *i* is ranged from [1, 2, 3] for single support phase, when *i* is 1, it represents the standing leg, when *i* equals to 2 it represents the swing thigh, and when *i* is 3 it is the swing shank,.

|  |  | （4） |
| --- | --- | --- |

where and denote the horizontal and vertical velocities of the centroid of the *i* th segment, respectively, and refers to the angular velocity of the *i* th segment.

*at page 10 line 197-201:*

During double support phase, to distinguish the differences from the single support phase, the corresponding joint angles are added with a subscript “*d*” after 1 and 2. $\theta_{1d}$ represents the angle between the trailing leg and vertical direction, while $\theta_{2d}$ denotes the angle between the leading leg and vertical direction, and $p_{2d}$ corresponds to the distance between the centroid of the leading leg and the hip.

*We hope the reviewer will satisfy with the revision.*

3. The authors used different cycles to estimate the cadence frequency. This is why the estimate results in one gait cycle, while in the data there may be multiple gait cycles. Although this can be understood, it is not properly reported in the methods section and should be clarified. The estimated data essentially represent an average. Instead, the authors should work on producing a time series of the trace according to the model for each gait cycle.

***Response:*** *We are grateful to the reviewer for this insightful comment and valuable suggestion. The reviewer is absolutely correct in identifying that our initial approach generated a single, averaged representation of a gait cycle, and this is a common and widely used approach in gait analysis. We agree that generating a time series for each individual gait cycle is a more rigorous approach, as it preserves the inter-cycle variability and provides a more dynamic assessment of the model's performance.* *In direct response to this comment, we have undertaken the following revisions to the manuscript:*

*Immediate Clarification in Methods:We have revised the Methods section to explicitly and accurately describe our approach. The revision are added at page 7 line 150-151 as follows:*

The input data from multiple gait cycles were time-normalized and averaged to produce a single, representative gait cycle for each participant.

*In addition,“Discussion”section has acknowledged this limitation at page 22 line 405-408. We formally incorporate the reviewer's excellent suggestion into our future research agenda. The revision are added at page 22 line 409-410 as follows:*

Additionally, the established predictive model assumes that the walking characteristics across each gait cycle are periodically identical. While human walking is random and varies considerably, an individual cannot repeat two identical steps [38]. This discrepancy contributed to the errors between the predictive model and the experimental results. Generating a full time-series of estimates for each gait cycle will enable more robust validation of the model’s performance across dynamic.

*We hope the reviewer will satisfy with the revision.*

4. The metrics computed are good but not sufficient. After being able to estimate the data, the authors should also use Statistical Parametric Mapping (SPM), which is freely available as software packages in both MATLAB and Python.

***Response:*** *Thank you for this valuable suggestion. We acknowledge the strengths of Statistical Parametric Mapping (SPM) for performing statistical comparisons. Our work focuses on model fitting and reconstruction of gait kinetics, where the primary objective is to estimate continuous kinetic signals from easy measured input data and to evaluate the numerical accuracy of these reconstructed signals. The corresponding revision are as follows:*

*At page 16 line 314-318 as:*

Statistical comparisons between predicted and experimentally collected signals were performed using one-dimensional Statistical Parametric Mapping (SPM) via the open-source SPM1D toolbox for MATLAB (https://spm1d.org/index.html) at a significance level of α= 0.05. Clusters where SPM{t} exceeded the corresponding critical threshold (t*) were considered statistically significant, and that indicates the predicting model is invalid.

*The corresponding analyzing results were added to each parameter predicted:*

*At page 17 line 331-335 as:*

Fig. 4. Measurement of the hip angle and knee angle in the sagittal plane of one subject. The horizontal axis of the gait cycle is determined on the basis of the left foot. The orange red dashed line indicates the critical thresholds (t* = 168.662, α = 0.05). Regions of the gait cycle for which SPM {t} exceeded the critical threshold are considered as the predicting model is invalid.

*At page 18 line 348-352 as:*

Fig. 5. The GRF across the entire gait cycle. The dashed line denotes the model-predicted value. The shadow region is composed of 8 walking trials of one subject. The orange red dashed line indicates the critical thresholds (t* = 250.126, α = 0.05). Regions of the gait cycle for which SPM {t} exceeded the critical threshold are considered as the predicting model is invalid.

*At page 19 line 366-370 as:*

Fig. 6. The joint moment across the entire gait cycle of one subject. The measurement moment in the figure is obtained from the walking trials of the corresponding subject in the experiment. The orange red dashed line indicates the critical thresholds (t* = 334.678, α = 0.05). Regions of the gait cycle for which SPM {t} exceeded the critical threshold are considered as the predicting model is invalid.

*Thank you again for raising this enlightening point.*

5. In the comparison table, the authors should expand the review to include methods based on EMG and IMU signals, which are wearable and represent a good framework for estimation. From this perspective, they could revise their work by considering the following manuscripts:
   • Scattolini, Mara, et al. "Inertial Sensing for Human Motion Analysis: Enabling Sensor-to-Body Calibration Through an Anatomical and Functional Combined Approach." IEEE Transactions on Neural Systems and Rehabilitation Engineering (2025).
   • Mengarelli, Alessandro, et al. "Myoelectric-Based Estimation of Vertical Ground Reaction Force during Unconstrained Walking by a Stacked One-Dimensional Convolutional Long Short-Term Memory Model." Sensors24.23 (2024): 7768.

***Response:*** *We sincerely thank the reviewer for this valuable suggestion. We fully agree that incorporating the latest estimation methods based on wearable sensors— particularly IMUs and EMGs will further improve the performance of the proposed method. The revision has been added to the revised manuscript at page 22 line 421-423 as follows:*

Future work will explore synergies with wearable technologies including advanced inertial measurement units [39] and electromyographic-driven models [40].

[39].Mara S, Andrea T, Federica V, et al. Inertial sensing for human motion analysis: Enabling sensor-to-body calibration through an anatomical and functional combined approach. IEEE Transactions on Neural Systems and Rehabilitation Engineering, 2025:33: 1853 - 1862.

[40].Mengarelli A, Tigrini A, Scattolini M, et al. Myoelectric-Based Estimation of vertical ground reaction force during unconstrained walking by a stacked One-Dimensional convolutional long Short-Term memory model. Sensors, 2024, 24(23): 7768.

*We hope this approach is acceptable. Thank you again for raising this point.*
